# Challenges and promising solutions to engaging patients in healthcare implementation in the United States: an environmental scan

**DOI:** 10.1186/s12913-023-10315-y

**Published:** 2024-01-04

**Authors:** Eva N. Woodward, Andrea Isabel Melgar Castillo, Gala True, Cathleen Willging, JoAnn E. Kirchner

**Affiliations:** 1 VA Center for Mental Healthcare and Outcomes Research, 2200 Fort Roots Drive, Building 11, North Little Rock, AR 72114 USA; 2 Department of Psychiatry, University of Arkansas for Medical Sciences, 4301 W Markham St, Little Rock, AR 72205 USA; 3 Graduate School, University of Arkansas for Medical Sciences, 4301 W Markham St, Little Rock, AR 72205 USA; 4 South Central Mental Illness Research Education and Clinical Center, Southeast Louisiana Veterans Health Care System, 2400 Canal St, New Orleans, LA 70119 USA; 5 Section on Community and Population Medicine, School of Medicine, Louisiana State University, 2400 Canal St (11F), New Orleans, LA USA; 6 Pacific Institute for Research and Evaluation, 851 University Boulevard, Suite 101, Albuquerque, NM 87106 USA; 7 Behavioral Health Quality Enhancement Research Initiative (QUERI), Central Arkansas Veterans Healthcare System, 2200 Fort Roots Drive, Building 11, North Little Rock, AR 72114 USA

**Keywords:** Patient engagement, Patient involvement, Implementation, Healthcare disparities, Community Engagement, Integrated knowledge translation, Participatory

## Abstract

**Background:**

One practice in healthcare implementation is patient engagement in quality improvement and systems redesign. Implementers in healthcare systems include clinical leadership, middle managers, quality improvement personnel, and others facilitating changes or adoption of new interventions. Patients provide input into different aspects of health *research*. However, there is little attention to involve patients in *implementing* interventions, especially in the United States (U.S.), and this might be essential to reduce inequities. Implementers need clear strategies to overcome challenges, and might be able to learn from countries outside the U.S.

**Methods:**

We wanted to understand existing work about how patients are being included in implementation activities in real world U.S. healthcare settings. We conducted an environmental scan of three data sources: webinars, published articles, and interviews with implementers who engaged patients in implementation activities in U.S. healthcare settings. We extracted, categorized, and triangulated from data sources the key activities, recurring challenges, and promising solutions using a coding template.

**Results:**

We found 27 examples of patient engagement in U.S. healthcare implementation across four webinars, 11 published articles, and seven interviews, mostly arranging patient engagement through groups and arranging processes for patients that changed how engaged they were able to be. Participants rarely specified if they were engaging a population experiencing healthcare inequities. Participants described eight recurring challenges; the two most frequently identified were: (1) recruiting patients representative of those served in the healthcare system; and (2) ensuring processes for equitable communication among all. We matched recurring challenges to promising solutions, such as logistic solutions on how to arrange meetings to enhance engagement or training in inclusivity and power-sharing.

**Conclusion:**

We clarified how some U.S. implementers are engaging patients in healthcare implementation activities using less and more intensive engagement. It was unclear whether reducing inequities was a goal. Patient engagement in redesigning U.S. healthcare service delivery appears similar to or less intense than in countries with more robust infrastructure for this, such as Canada and the United Kingdom. Challenges were common across jurisdictions, including retaining patients in the design/delivery of implementation activities. Implementers in any region can learn from those in other places.

**Supplementary Information:**

The online version contains supplementary material available at 10.1186/s12913-023-10315-y.

## Background

One emerging practice within implementation science is to engage patients or consumers of health care in implementing changes, system redesign, or quality improvement to ensure healthcare service delivery is more patient-centered [1]. Currently, patients provide input into different aspects of health *research*. As examples, patients make invaluable contributions to developing interventions, designing participant study recruitment approaches, and disseminating findings [2, 3]. However, there has been little attention on how to involve patients or the public in the process of implementing interventions in healthcare systems. It is possible patient engagement in design/delivery of implementation might result in strategies targeting an increase in patient buy-in or demand for certain interventions, which is one hypothesized mechanism to reduce health care inequities. Tailoring interventions or implementation for local context—which patients can help guide as end users with lived experience—might be another essential mechanism to reducing healthcare inequities, and ignoring their potential contributions might exacerbate disparities [4].

Research on engaging patients in implementation activities in the U.S. is known as *patient engagement in design/delivery of implementation*, quality improvement, and systems redesign, [5] participatory implementation science, [6] or community-engaged dissemination and implementation [1]. For the present analysis, we are not using the phrase patient engagement to refer to patient activation in their own health care, although it is possible that patient engagement in implementation might lead to strategies focused on patient activation. *Implementers* in healthcare systems can include clinical leadership, middle managers, quality improvement personnel, or other people facilitating changes or adoption of new interventions.

Although implementers can use multilevel strategies to increase adoption of interventions among providers, clinics, and healthcare systems (e.g., training providers, changing physical infrastructure), [7] engaging patients in implementation activities is currently rare. Implementers can engage patients in implementation activities in many ways [8]. A consensus process with implementation experts highlighted five categories of patient engagement in implementation activities: (1) involve patients/family members in implementation efforts; (2) intervene with patients to enhance uptake and adherence; (3) prepare patients to be active participants in their care (akin to “patient activation”); (4) increase demand among patients, so they ask for the intervention; and (5) use mass media to spread awareness of the intervention [9]. The expert panel suggested activities for including patients in the pre-implementation phase or training them in the intervention or how to advocate for it. Other suggestions were to increase awareness to enhance patient demand for the intervention (e.g., what pharmaceutical companies do) and prepare them to ask questions about their individual care, thus increasing pressure for adoption of the intervention.

In the U.S., despite increasing requirements by payers and organizations to engage patients in implementation or quality improvement, [10–12] the practice appears to be uncommon [13, 14]. Other countries, such as Canada and the United Kingdom (U.K.), have longer histories of “patient and public involvement” in healthcare implementation activities, supported by funding, personnel, training, and expectations to do so in their healthcare systems [15–20]. Yet, for U.S. settings, little detail is published on these strategies, and there are few well-documented examples in routine healthcare service delivery, despite the unique economic, political, regulatory, and social contexts of U.S. health care [19–22]. We need greater detail on how patients are engaged in implementation activities in U.S. healthcare systems, common challenges, and ways implementers overcome those challenges.

Limited work on this topic in the U.S. shows preliminary benefits, yet patient engagement in implementing routine healthcare service delivery is not adequately supported across the nation’s healthcare systems. For example, early work showcasing patient engagement in implementation activities has resulted in patient-centered systems redesign in healthcare settings, [23] greater uptake of effective interventions in community settings, [24] including those experiencing health care disparities in access and utilization, [25] improved patient health, [25] and increased sustainment of interventions [26]. Proposed mechanisms from early work suggest that the aforementioned outcomes improve due to increasing relevance or fit of interventions and implementation strategies to real-world settings, using patient-driven solutions to challenges, and/or building the capacity of all involved for implementation and sustainment [27]. Similarly, the Patient-Centered Outcomes Research Institute (PCORI) in the U.S. funds *implementation research* that engages patients and other end users but does not fund sustained implementation efforts directly in healthcare systems. The U.S. Agency for Healthcare Research and Quality produces some guidance on one specific strategy (e.g., patient advisory councils), [28] yet, does do not provide funding for these activities in U.S. healthcare systems.

We also lack enough details or standards of optimal patient engagement in implementation activities in the U.S., limiting the knowledge other implementers could use in their efforts [22]. It is unclear whether implementers in U.S. healthcare settings face similar challenges or can enact similar solutions as those in other countries. As a first step to developing an approach to engaging patients in healthcare implementation activities, we piloted an environmental scan methodology derived from business literature. As described in Methods, we reviewed or “scanned” three data sources meeting industry standards for health services research. We also documented details of what, how, and in what contexts implementers engaged patients in implementation activities [29] in U.S. healthcare settings, describing challenges and solutions.

## Methods

### Design and procedures

Environmental scanning originated in the business field to document trends to assist in data-driven planning [30, 31]. We applied this approach to patient engagement in implementation in healthcare contexts. The goal of this approach is not to produce a comprehensive understanding but to capture a snapshot of trends through a variety of data. Our environmental scan also illustrates which cases focused on healthcare disparities or inequities. The conduct of the scan required openness, the anticipation of new gaps, and a willingness to revise existing knowledge based on the data reviewed [32]. This study is under regulation by the Central Arkansas Veterans Healthcare System Institutional Review Board, which deemed it not human subjects research. Thus, participants interviewed were not formally consented, but before deciding whether to participate, they were apprised of the study’s rationale and told the information they provided would be de-identified in dissemination.

### Inclusion and exclusion criteria

We set the following *a priori* inclusion criteria for all data sources in this scan: (1) patients were engaged in implementation, service delivery, systems redesign, or quality improvement of an intervention, and could be engaged in dissemination if it was used as an implementation strategy; (2) the focus of the implementation effort was a health condition; (3) service delivery was conducted partially or completely in a U.S. healthcare setting; and (4) the study or activity used descriptive, qualitative, quantitative, or mixed-methods research. A case was excluded if the focus was: (1) patient engagement only in research, not implementation; (2) patient engagement in direct care that may only result in changes for one individual’s health; (3) guideline or instrument development, theoretical or conceptual articles, literature review, or protocol papers; or (4) outside the U.S.

### Data sources

**Fig. 1:**
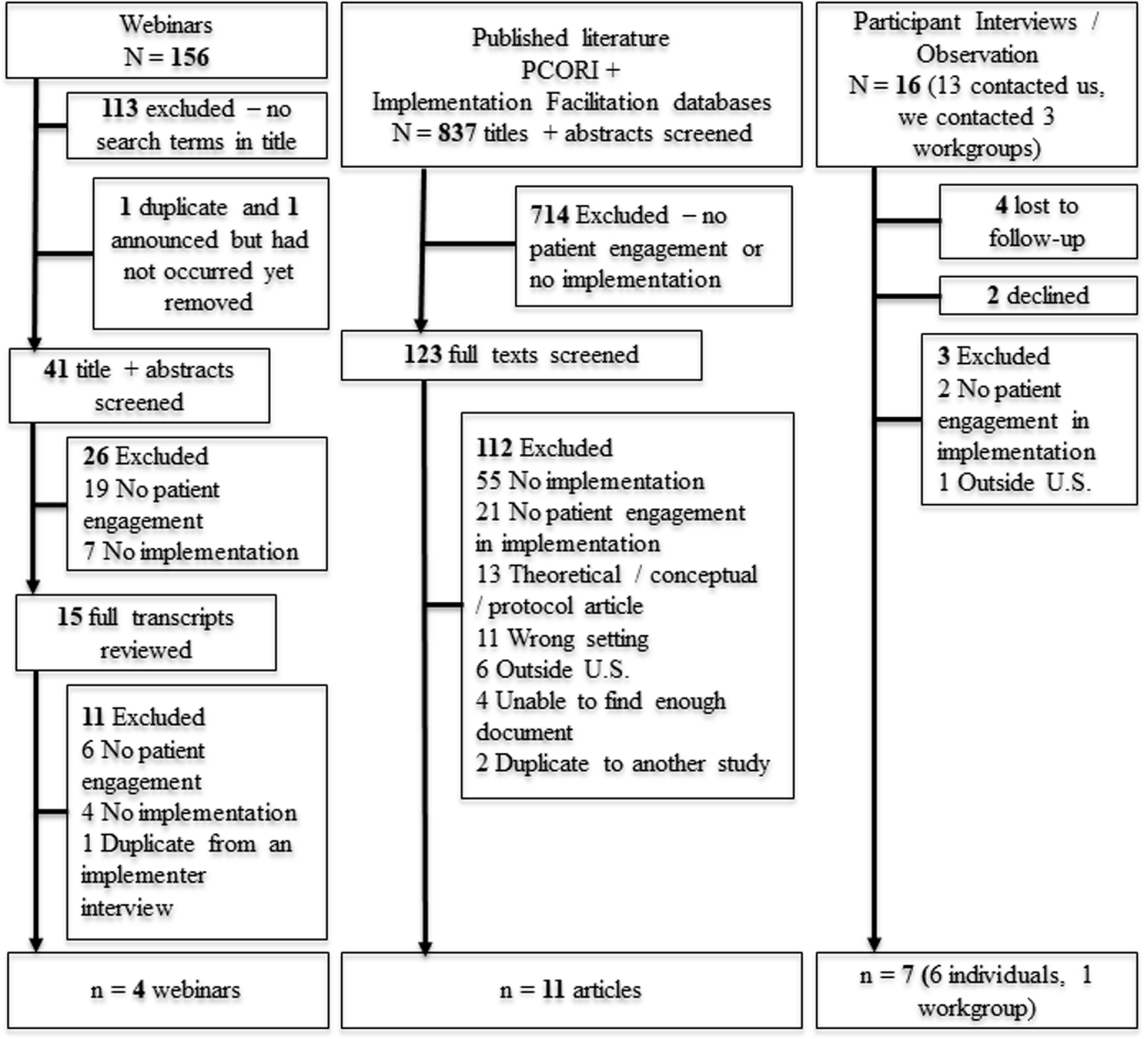
Flowchart of data collection for environmental scan of patient engagement in U.S. healthcare implementation activities

We captured data from three data sources for this environmental scan. We systematically searched existing and recent databases for published literature (rather than conducting a new systematic review) and webinars and interviewed implementers individually or observed them in a workgroup setting (i.e., *interview participants)*. We completed our first round of data collection in 2018–2019 and updated literature and webinar searches in 2022. Figure 1 describes our systematic search and recruitment process.

#### Literature

We drew from two existing databases of articles: The PCORI Health Research Literature Explorer [33] and the Implementation Facilitation Literature Collection created by the VHA Behavioral Health Quality Enhancement Research Initiative [34]. Both databases were collections of articles populated using (1) systematic review methods, i.e., they applied a rigorously developed search strategy using a wide variety of search terms including keywords and MeSH terms; (2) applied the search in industry-standard, indexed search engines (i.e., PubMed/MEDLINE, CINAHL, and Thompson Scientific Web of Science); and (3) conducted a two-stage screening process applying relevant inclusion and exclusion criteria [33, 34]. Thus, instead of conducting a new systematic review, we relied on two databases that feature results of systematic review methods (i.e., articles). Then, within each database, we narrowed our search for articles more closely to our inclusion criteria—patient engagement (i.e., PCORI database) or changes in healthcare service delivery (i.e., Implementation Facilitation database). We imported selected articles into a systematic review software [35].

Updated through May 2021, the PCORI database contained literature focused on patient engagement in health research [33]. We searched for articles under the categories “dissemination” or “translation” phases of research, then under the categories “patients” and/or “caregivers” for patient involvement, and screened all titles and abstracts for patient engagement in healthcare implementation activities.

The second database was produced through a systematic review by Ritchie and colleagues [34] of peer-reviewed articles on Implementation Facilitation, an implementation strategy that includes relationship building, interactive problem solving, and other participatory approaches for supporting change. This database contained literature in any context (including outside VHA) from 1996 to 2020. We chose to use this Implementation Facilitation literature database because it aligned with our scan’s focus on making changes in healthcare service delivery or implementation in clinical care instead of only research studies. We searched and screened every abstract and title for patient engagement without narrowing by search terms.

#### Webinars

We searched webinars from the U.S. Veterans Health Administration (VHA) Health Services Research and Development archive, as this is the only database we identified of *webinars* with examples of implementation or changes in U.S. healthcare service delivery. We systematically searched these terms in webinar titles archived from the earliest possible date (January 1, 2016) through May 24, 2022:

1. “patient”, “user”, “client”, “patient”, “veteran”, “caregiver”, “partner”, OR “family” AND
2. “engag*”, “participat*”, “involv*”, OR “consult*”.

#### Interview Participants

We recruited implementers as interview participants—healthcare professionals who engaged patients in design/delivery of implementation activities and facilitated change in healthcare service delivery. To recruit a convenience sample, we distributed a flyer via email invitation sent to U.S. professional organizations with an implementation science affiliation (e.g., Implementation Research Institute, Mentored Training for Dissemination and Implementation in Cancer) and conducted outreach through Twitter. We engaged potential participants in snowball sampling, asking them to suggest other candidates for participation [36]. Through this approach, we contacted three workgroups we were alerted to by potential participants. After one round of interviews and analysis, we had not saturated results. Thus, we conducted a second round until we interviewed enough participants to saturate results [37].

Based on participant preference, we sent an electronic survey with open-ended text boxes for self-administration or conducted 45-minute telephone interviews with the same questions. As described in Additional file 1, questions included: “*What structures or tools or practices have you used to do this work? What are some problems you encountered in engaging consumers in implementation? How did you solve them?”* In one case, we were able to observe a workgroup of participants and coders used the same questions as in the survey/interview and populated responses into a coding template.

### Data extraction using a coding template

We extracted and categorized data from all sources using a coding template informed by two theoretical frameworks chosen because of their content relevance to the research questions about patient engagement, implementation science, and health inequities (see Additional file 2) [38]. Goodman and Sanders Thompsons’ stakeholder engagement framework depicts intensity levels at which researchers engage patients, including methods not truly engaged to highly engaged participation [39]. The Health Equity Implementation Framework highlights domains relevant to implementation and healthcare disparities, such as the intervention being implemented, who is involved (recipients), and societal context [40]. We assessed whether participants mentioned specific disparities among patients. Coders 1 and 2 (AIMC, ENW) independently extracted a random sample from all data sources and met for consensus until 80% agreement was met between them, then extracted the remaining data independently. In some data sources, we identified more than one “case” or example of patient engagement in implementation activities and extracted each case.

### Data integration and analysis

We merged all cases, or specific examples, of patient engagement in implementation activities from all three data sources into a final dataset. Our final dataset included specific examples with descriptions of each and patient engagement level according to Goodman and Sanders Thompsons’ framework (the latter was determined by Coders 1 and 2 and a third coder [JEK or CW] resolved disagreements). Next, coders synthesized descriptions of all data into a matrix of Health Equity Implementation Framework domains. They then reviewed each case for recurring challenges, grouping them into themes with 100% agreement, and extracted every potential solution mentioned for those challenges.

## Results

### Data description

We included 22 cases: four webinars, 11 literature articles, and seven interview participants in our final sample (see Fig. 1). From these, we identified 27 examples of implementers (from webinars, articles, or interviews) using patient engagement in healthcare service delivery implementation activities. All originated from the past decade. See Table 1 for descriptive information about what innovations were being implemented, in which settings, and for whom, according to the Health Equity Implementation Framework. One note is that, in describing elements of patient engagement in implementation activities, implementers rarely specified if they were engaging a population experiencing healthcare inequities.

**Table 1:** Descriptions of all patient engagement in U.S. healthcare implementation activities from environmental scan, described using health equity implementation framework domains^a^

Data Source	Innovation^c^	Patient Recipients	Provider/ Staff Recipients	Inner Context and Outer Context	Societal Context^b^	Population with Health Disparity?^c^
Articles from Literature Review						
Angstman 2009 [41]	Several. Some evidence based (e.g., depression care management). Most were process improvements to clinical care (e.g., Saturday clinic, internet portal for patients)	Primary care patients or parents of patients	Primary care providers and staff	One primary care clinic; Network of clinics in Minnesota	Not specified	Not specified
Norman 2013 [42]	Several. Evidence based (e.g., hypertension home monitoring, colorectal cancer screening)	Several	Healthcare providers	Unspecified clinics in Colorado; Single U.S. healthcare system	Not specified	Rural and frontier communities
Pérez Jolles 2017 [43]	Parent activation to increase mental health service engagement for youth. Evidence informed.	Parents of youth with mental health needs	Mental health care staff and directors	One mental health clinic in North Carolina	Sociopolitical: (+/-) Values of immigrant cultures affect perception of U.S. health care	Latino families who were less likely to access mental health services. Spanish speaking
Tapp 2017 [44]	Shared decision- making toolkit. Evidence based.	Patients with asthma	Healthcare providers	Many types of clinics; Practice based research network and advanced Medicaid network in North Carolina	Not specified	Yes, not specified
English 2018 and Dickinson 2020 (same study but articles reported on two separate patient engagement activities) [45, 46]	Cardiovascular prevention strategies (e.g., improving clinical management of aspirin use, blood pressure control). Evidence based.	Primary care patients	Primary care providers	211 primary care clinics in two U.S. states (Colorado and New Mexico)	(+/-) tracked % of adults who were uninsured, living below poverty level, local unemployment rate, median income, primary care provider shortages; (+) Collaborative funded by Agency for Health care Research and Quality	Not specified. Reported clinics were in diverse racial and socioeconomic patient serving places
Barger 2019 [47]	Prescribing primary prophylactic colony stimulating factors. Evidence informed.	Patients receiving myelosuppressive chemotherapy	Physicians	45 clinics in National Cancer Institute Community Oncology Research Program	Not specified	Not specified
Browne 2020 [48]	Transitions of kidney disease care. Evidence base not specified.	Patients with advanced chronic kidney disease	Kidney specialists	One hospital and several smaller clinics	Single healthcare system in Pennsylvania	Not specified
Pandhi 2020 [49]	Several, e.g., depression care management, patient service centers, Saturday primary care clinics - some evidence based, most were greater process improvements to clinical care	Patients	Physicians, nurses, QI personnel, clinic managers, medical assistants	2 primary care clinics in different healthcare systems across U.S.	(+/-) One clinic urban, one clinic rural (+) increasing emphasis on patient engagement by payers (e.g., CMS)	Not specified
Pekmezaris 2020 [50]	Home telemonitoring for chronic obstructive pulmonary disease	Patients with chronic obstructive pulmonary disease	Pulmonologists, primary care physicians, geriatricians, respiratory therapists	Pulmonary rehabilitation centers in New York metropolitan area	Not specified	African American and Hispanic patients
Gesell 2021 [51]	Post-acute stroke transitional care model. Evidence based	Patients with stroke and transient ischemic attack	Healthcare providers, social service providers, post-acute care coordinator	40 hospitals in North Carolina	Not specified	Variety of people from urban and rural areas, varied income and education
Webinars						
Fehling et al., 2016 [52]	Several (e.g., traumatic brain injury interventions). Evidence base not specified.	VHA patients	Not specified	Research Centers of Innovation; VHA healthcare system	Not specified	Yes, several
LaChappelle et al., 2017 [53]	Several (e.g., pain management). Evidence base not specified.	VHA patients	VHA providers	Geographic regions: Denver, Houston, Iowa City catchment areas; VHA healthcare system	Sociopolitical: (-) Unsure how to disseminate information to policymakers	Yes, several
Asch 2018 [54]	HIV testing. Evidence based.	Patients at high risk for HIV	Primary care providers in VHA	Primary care clinics in selected regions in VHA healthcare system	Sociopolitical: (-) Stigma about HIV risk behavior makes it harder to reach people at high HIV risk	Not specified
Elwy 2018 [55]	Several. Evidence based not specified.	Several	Several	Implementation research centers; VHA healthcare system	Not specified	Not specified
Participant Interviews / Observations						
Participant Interview 1	Several (e.g., blood pressure monitoring, colorectal cancer screening). Evidence based.	Several	Clinic and quality improvement leaders; Community health advocates	12–26 clinics in in Washington, California, Oregon; Single U.S. integrated health care system, Federally Qualified Health Center system, or two Medicaid managed care insurance plans	Economic: (-) Challenges working with insurance payers	Low income
Participant Interview 2	Trauma psychotherapy. Evidence based.	VHA patients exposed to traumatic events	Mental health providers, clinic administrators	Several mental health clinics in one VHA hospital in Massachusetts	Not specified	Not specified
Participant Interview 3	Notification letter informing parents their children were being placed on a treatment waitlist. Not evidence based.	Parents of children with mental health concerns (children were patients)	Mental health providers, administrative assistant	One outpatient mental health clinic; Private university hospital in New York	Economic: (-) Having public insurance made it hard to find other providers, so waitlist notification was even more upsetting to families because they did not have other options. (+) Implementation initiative funded by a health foundation because it would not otherwise be billable by insurance	People of color
Participant Interview 4	Diabetes self-management program. Evidence based.	Latino diabetes patients or those at risk for diabetes	Clinic managers, community health worker, providers	One community clinic in New Mexico; No larger healthcare system	Economic: (+) external funder that valued community engagement	Immigrants, Latino ethnicity, mainly Spanish speaking, mainly low income
Participant Interview 5	Several. Evidence base not specified.	VHA patients	VHA providers and staff	Seven community clinics and three larger hospitals in California; VHA healthcare system	Physical structures: (-) difficult for some to get to meetings due to lack of affordable or easy transport	People of color
Participant Interview 6	Several (substance use or mental health focus). Evidence base not specified.	VHA patients in recovery who used VHA addiction and/or mental health services	VHA providers and staff	Selected clinics in VHA healthcare system	Not specified	Primarily Black and Latino patients
Participant Interview 7	Several. Evidence base not specified.	VHA patients	Not specified	Hospitals and clinics; VHA healthcare system	Sociopolitical: (+) U.S. military culture facilitates teamwork. Economic: (-) Limited financial resources	Not specified

Note. Examples listed in chronological order. *a*. Domains are from the Health Equity Implementation Framework (e.g., innovation, societal influences), although one domain, the clinical encounter, is omitted because no factors in this domain were identified in data collection. *b*. Societal influences could include: Sociopolitical factors (e.g., laws, policies), Economic factors (e.g., insurance), or Physical Structures (e.g., the built environment, signs, location of health care). (+) = factor was a facilitator or strength for implementation. (-) = factor was a barrier or deterrent for implementation. *c*. Health disparity population is defined as a group that experiences disparities in health outcomes or access to or quality of health care in within a certain health condition (e.g., HIV). ^c^We denoted the level of research evidence for each innovation, or whether the evidence base was unknown. Evidence informed means it has preliminary evidence for some outcomes, but not robust enough research to draw strong conclusions about health outcomes

### Examples and intensity levels of patient engagement in implementation activities

We described how implementers operationalized patient engagement in implementation activities, sorting them by patient engagement intensity level according to Goodman and Sanders Thompson’s framework in Table 2.

**Table 2:** Examples of patient engagement in U.S. healthcare implementation activities by intensity of patient engagement identified in an environmental scan (Goodman and Sanders Thompson, 2017)

**Data Source**	**Patient Engagement Level: Outreach Example (*n* = 0)**^a^ Implementers develop, implement, and evaluate strategies to reach target populations. Patients of target population can be engaged as advisors and make key connections. (Non-participation)
n/a	No examples identified
**Data Source**	**Patient Engagement Level: Education Example (*n* = 0)** Implementers try to educate patients about a topic (e.g., gain audiences for education sessions). (Non-participation)
n/a	No examples identified
**Data Source**	**Patient Engagement Level: Coordination Examples (*n* = 13)** Implementers gather patients to inform elements of a study or activity. Patients give feedback, which informs implementers’ decisions, but it is the implementers’ responsibility to design and implement the study with no help expected from patients. Implementation activities are strengthened through community outreach, and results are disseminated through community groups and gatekeepers. (Symbolic participation)
Angstman 2009 (Literature article) [41]	Parents of child patients were engaged in a patient advisory group with an interest in improving healthcare for a clinic.
Fehling 2016 (Webinar) [52]	Coordinated a patient engagement group described below in LaChapelle [53].
LaChapelle 2017 (Webinar) [53]	Coordinated a recurring, monthly patient engagement group that consults with researchers (some of whom study implementation). Researchers presented to patient engagement group and get feedback on their research at the idea generation stage through study completion, all the way to the dissemination stage.
Perez Jolles 2017 (Literature article) [43]	For children’s mental health efforts, a parents advisory group met every three months provided input on implementation research protocols, implementation decisions in the context of a study, interpreted findings, and suggested next steps.
Tapp 2017 (Literature article) [44]	Engaged up to sixteen patients on a patient advisory board who participated in one or more of implementation study phases: study design, approving protocols, intervention implementation, study management, data analysis, or dissemination.
Asch 2018 (Webinar) [54]	Involved a patient representative on a grant-funded implementation project series, ranging from identifying current variation in implementation practice to implementing an innovation.
Elwy 2018 (Webinar) [55]	Involved patients through steering committees and advisory boards in guiding development of implementation research and/or disseminating findings from implementation research.
Participant Interview 1	Interviewed and selected patients to serve as advisors and co-investigators on a patient advisory group for quality improvement and implementation initiatives.
Participant Interview 2	Engaged as patients in adapting use of evidence-based psychotherapy in routine care and after treatment, providing feedback via qualitative interviews on feasibility, acceptability, and suggested adaptations of the psychotherapy.
Participant Interview 7	Organized patients who were involved in were implementation studies to attend a national conference and present at talks and station an informational table. The patients’ goals at the conference were to disseminate findings, answer other researchers’ questions in group discussions, and market the value of patient patients in implementation research to more researchers.
Pandhi 2020 (Literature article) [49]	Clinic staff engaged patients in quality improvement activities: 1) Surveyed current patients about preferences for the timing of lab work vis-à-vis scheduled appointments and used their preferences to rework clinic flow. Then, re-surveyed patient satisfaction after changes were implemented. 2) Invited over 100 patients with asthma to a lunch and learn at which the new asthma plans would be explained and patient feedback solicited.
Participant Interview 3	For children’s mental health services, an outside agency helped one clinic form a clinic-family advisory council. This council engaged in workshops to brainstorm projects for improving healthcare delivery at their clinic. Once they selected projects to improve care, clinic staff carried out most steps to implement changes and reviewed these with families from the council periodically. Everyone had input and the clinic director had final approval.
Dickinson 2020 (Literature article) [46]	Clinics created patient and family advisory councils serving each clinic. Main research collaborative trained practice facilitators to work with clinics to support formation and use of patient and family advisory councils.
**Data Source**	**Patient Engagement Level: Cooperation Examples (*n* = 5)** Implementers ask patients for help instead of only asking for advice. There is some activity on the part of patients in aspects of the project, including recruitment, implementation of interventions, measurement, or interpretation of outcomes. Patients are ongoing partners in decision-making. Patient understanding of implementation and its potential importance is enhanced. (Symbolic participation)
Tapp 2017 (Literature article) [44]	Patient advisory board gave input on dissemination strategies that facilitated implementation of toolkits for patients with asthma.
Elwy 2018 (Webinar) [55]	Patients disseminated information about implementation studies and results to key patient groups and policymakers through social media and conference presentations.
English 2018 (Literature article) [45]	Boot Camp Translation with community members and healthcare professionals involving a one-day retreat, 4-6 conference calls, and 3-4 in-person meetings. They discussed complex health topics and clinical guidelines and decided which ones to focus on for patient education, then produced a set of locally relevant actionable messages and materials to be distributed to patients.
Participant Interview 7	29 patients involved in dissemination of implementation research findings at a national research conference.
Participant Interview 5	Patients who served on a central patient-only advisory board acted independently on behalf of the board by participating as a patient representative on many hospital-wide committees.
**Data Source**	**Patient Engagement Level: Collaboration Examples (*n* = 7)** Implementers and patients are actively involved in the design and implementation of a study or activity and interpretation of findings. All benefit from working together, including increased capacity of patients to engage in implementation activities. Patients collaborate in decision-making and resource allocation with an equitable balance of power that values their input. (Engaged participation)
Tapp 2017 (Literature article) [44]	Caregiver advocates (proxies to patients) attended project meetings, gave input into patient-centered approaches, assisted with data interpretation and analysis, contributed to dissemination strategies, and were involved in advocacy and policy development.
Participant Interview 1	Patient advisory group members served as co-investigators on implementation projects.
Participant Interview 6	Patients co-created an advisory board partnership with implementation researchers. Patients were research partners, completed human ethics training, listed as key personnel, wrote letters of support for grants, developed name, mission, and purpose of the board, and helped to design, operationalize, and complete implementation research as needed.
Barger 2019 (Literature article) [47]	An external stakeholder advisory group was assembled to inform each phase of a research trial from planning and design to implementation and dissemination. They convened during two web conferences, two patient partner-specific web conferences and one in-person meeting held in conjunction with a larger group meeting. These interactive meetings facilitate regular communication about progress and collaborative problem solving. The team also reached out to specific people over email to request feedback on issues.
Browne 2020 (Literature article) [48]	Patients and family members worked as co-investigators in the development and implementation of a 5-year implementation study. Patients and family members were considered true team experts and full partners, attending regular research team meetings and not serving only in an “advisory” role, but being compensated, providing input on the study design and conduct, and being involved in analyses and dissemination.
Pekmezaris 2020 (Literature article) [50]	Created a community advisory board for implementation of a new intervention, including patients, nonprofessional caregivers, experts in health and social disparities, clinicians, and patient advocates. The role of the community advisory board was to advise the implementation team on all aspects of design, implementation, evaluation, and dissemination over time. This led to discussions on adaptation, usability, and program satisfaction and ensured that the conduct of the project remained patient-oriented.
Gesell 2021 (Literature article) [51]	Created a statewide stakeholder committee to contribute to design, conduct, and dissemination of findings of a multicenter pragmatic clinical trial. The committee added, shaped, and refined intervention components and all patient- and provider-facing materials.
**Data Source**	**Patient Engagement Level: Patient-Centered Example (*n* = 1)** Patients, caregivers, or advocacy groups assume responsibility for priority setting for activities, control design and implementation of activities, and manage interpretation and dissemination of findings. Implementers use expertise to move these things along, but patients make all major decisions. Systems are in place for patient participation at all engagement levels. Patients can collaborate with equitable balance of power for governance and strong level of accountability to their community. (Engaged participation)
Participant Interview 4	Information gathered in preliminary participatory research with patients was then used by patients to implement a new diabetes self-management program at a community clinic. Patients who worked at the clinic also engaged patients through an advisory board and community organization to determine delivery of the program.
**Data Source**	**Patient Engagement Level: Community-Based Participatory Research (CBPR) Example (***n*** = 1)**: CBPR is the population health approach to the “patient-centered engagement” level. Principles of CBPR are applied to implementation, including trust among partners, respect for each partner’s contributions, mutual benefit, and a community-driven collaboration with equitable and shared decision-making. (Engaged participation)
Norman 2013 (Literature article) [42]	Community advisory council consisting of patients developed a CBPR approach to develop and test messages and dissemination strategies for several healthcare issues. The council considered potential projects presented by researchers from the nearby university and selected topics based on community priorities, potential for funding, and potential impact on their community. The council helped with data analysis, interpretation of results, and dissemination of findings. The council was joined by health professionals, health department representatives, hospital administrators, and patients with the health condition of interest.

Note. *a.* Data listed in chronological order and categorized according to Goodman and Sanders Thompson’s (2017) stakeholder engagement framework. We slightly adapted the framework to be about implementers and patients rather than researchers and community stakeholders, and the activities to be about implementation of healthcare service delivery rather than research

Half of the examples featured implementers engaging patients in coordination, a medium-intensity level of patient engagement. Coordination typically involved convening patients in groups for the provision of unidirectional feedback, such as using recurring, monthly patient engagement groups that consulted with researchers (some of whom studied implementation) in the example by LaChapelle [53]. Sometimes patients were engaged individually, still at the “coordination” level, to provide unidirectional feedback, as showcased in the example in Participant Interview 2 in which patients completed surveys and interviews about how to adapt a psychotherapy after having exposure to the treatment. Between levels of intensity regarding engagement, sometimes the “how” patients were engaged were similar, yet processes or roles differed enough that patient engagement was made more meaningful. For example, many of the activities categorized under “coordination”—a lower intensity engagement activity—and “collaboration”—a higher intensity engagement activity—involved assembling patient advisory groups. Upon closer review, there were processes and roles in those activities we considered collaboration that signaled a deeper, longer, and more patient-centered approach, such as being involved in all phases of implementation (e.g., Browne 2020, literature article), [48] serving in roles with more power or voice (e.g., Participant Interview 1), and patients seeing how their input was incorporated (e.g., Gesell 2021, literature article) [51].

**Fig. 2:**
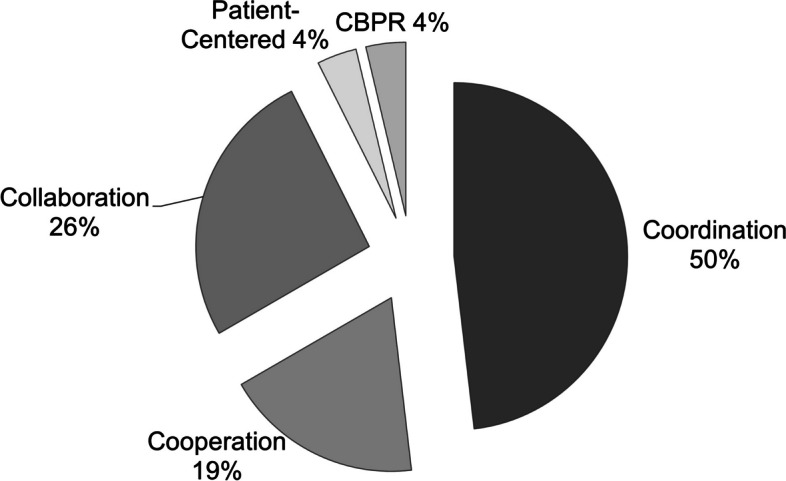
Percentage of patient engagement examples in U.S. healthcare implementation activities by intensity, classified according to Goodman and Sanders Thompson (2017). *Note*. CBPR = community-based participatory research. Starting at coordination and moving clockwise, the levels range from least intensive to more intensive patient engagement, ending with CBPR

The most intensive patient engagement activity (Norman 2013, literature article), also shown in Table 2, involved a community advisory council consisting of patients who set priorities for implementation (e.g., which disease to focus on), brought in healthcare professionals, and helped those professionals develop and test dissemination strategies for several healthcare issues. In this case, healthcare professionals assisted with an ongoing community initiative and patients have initial decision making capability, dictated priorities, engaged repeatedly over long periods of time about design/delivery for better implementation, and were compensated for their time [42]. As shown in Fig. 2, the remaining examples featured cooperating or collaborating with patients, representing incrementally more intensive levels of patient engagement than coordinating with them.

### Recurring challenges and solutions

We identified eight recurring challenges mentioned as barriers in at least two examples. Recurring challenges included: (1) Lack of processes to communicate equitably among all people involved; (2) issues recruiting patients who had a demographic makeup consistent with actual patients served across different intensity levels of engagement; (3) inadequate logistic, financial, or educational resources to successfully incentivize or carry out patient engagement activities; (4) difficulty retaining patients over time in implementation effort; (5) risk of tokenism: asking one member of a patient population to represent all patients from that population; (6) patients were not in suitable roles, and wanted to do something other than implementation, such as disseminate results or be actual research participants; (7) lack of feedback given to patients about results of their engagement; and (8) patients were engaged too late in the implementation effort for their engagement to be useful or realized.

In Table 3, we describe in more detail challenges mentioned and matched challenges with promising solutions extracted from data. In this text, we describe the two most frequently mentioned challenges and promising solutions in depth.

**Table 3:** Recurring challenges and promising solutions to engaging patients in U.S. healthcare implementation activities identified from an environmental scan

Recurring Challenge	Quotations or Examples from Data	Promising Solutions
1) Lack of processes to communicate equitably among stakeholders: modes of communication, informal / formal processes to feel comfort sharing and receiving feedback bidirectionally, a nonjudgmental atmosphere, and opportunities for all to speak and listen.	“We were encouraged to have families from low-income communities participate since they are usually underrepresented in our services, but that was challenging” (Participant Interview 3) Some patients were reluctant to participate in meetings with professionals(52)	• Human subjects’ ethics training or other courses (Participant Interview 6) • Rules of engagement to guide meetings (e.g., confidentiality; right to pass and not share) (LaChapelle, webinar, 2017) [53] • No acronyms (Fehling, webinar, 2016) [52] • Focus on practicality (Asch, webinar, 2018) [54] • Actively elicit patient feedback [56–58] • Provide orientation for patients [58] • Allow comments that are not “pro-healthcare” [58] • Group size < 15 members [58] • Explicitly state that patient experience is highly valued [57] • Empower patients to educate one another and implementers on topics [56]
2) Issues recruiting patients who had a demographic makeup consistent with actual patients served, across levels of engagement.	Patients engaged as partners can be overwhelmed by all the technical language providers and healthcare systems use (Participant Interview 5) Challenges encouraging patients to engage while also safeguarding them from being forced to overshare. (LaChapelle, webinar, 2017) [53]	• Accommodations to facilitate participation (e.g., childcare, transportation) [42] • Compensate patients [42]
3) Inadequate logistic, financial, or educational resources: Not having enough space, money, or knowledge to successfully incentivize or carry out patient engagement activities.	Some implementers mentioned logistic and financial barriers when engaging patients, including physical structures such as far distances to travel for an event. [59] Others mentioned educational barriers on behalf of staff and professionals, such as not having experience with quality improvement or patient advisory councils.(56)	• Ask for additional funding (Participant Interview 7) • Meetings held only as often as needed, no more [56, 58] • Limit to what is possible and do it well [43] • For lack of education, use multiple teaching methods to enhance knowledge (e.g., centralized trainer, online learning) [41]
4) Difficulty retaining patients over time in implementation effort	Implementers found that if they were to engage patients representative of patients affected by a particular health condition or treatment (i.e., overcoming challenge #1), they were then faced with how to retain those patients because of limited capacity demanded by the very health condition or treatment under study. One mentioned experiencing “tremendous turnover.” (Participant Interview 6)	• Recruit a *group* of patients to ensure engagement despite turnover (Participant Interview 6) • Experiment with different communication streams to figure out what is best (Implementer 3) [56, 57] • Alternate meeting times to enhance chances of attendance [57] • One-on-ones with patients, too [57] • Involve patients in conferences and: (a) arrange events needing little navigation from professionals; (b) plan for a “buddy” system; (c) host an orientation about the conference; and (d) create a flexible agenda for patients (Participant Interview 7)
5) Risk of tokenism: asking one member of a patient population to narrowly represent all patients from that population.	“If there is only one patient represented on a healthcare improvement issue, the staff might ask the patient to speak on behalf of all patients.” (Participant Interview 5)	• Diverse representation of patients (Fehling, Webinar, 2016) [52] • Allowing patients time and ability to become their own community (Fehling, webinar, 2016) [52] • Patients trained to ensure inclusive culture for others (Participant Interview 5)
6) Patients were not in suitable roles, wanting to do something other than implementation, such as disseminate results or be actual research participants	In one example, an implementer involved patients in adapting an intervention as it was being used in clinical practice, and some patients just wanted to receive treatment, rather than help change it, even after their initial consent to help implementers. (Participant Interview 2)	• Discuss pros and cons of engaging in implementing care versus routine care (Participant Interview 2) • Offer for patients to engage in other roles, e.g., feedback on study specifics, working on mission statements (Fehling, Webinar, 2016) [52] • “Trial and error, using a multi-pronged approach”: (a) basic information sheet about the effort, (b) initial meet and greet about the effort to discuss goals and answer questions, (c) assess patient buy in, and decision about participation, their fit, and discussions about “the ask.” (Participant Interview 1)
7) Lack of feedback given to patients: patients did not feel results of their engagement were being shared back with them.	Patients perceived it to be a problem because they were “not receiving feedback on whether anything changed as a result of their input” (Fehling, Webinar, 2016) [52]. One interesting note is this challenge was only mentioned in situations involving a coordination level of patient engagement. Because coordination efforts did not have patients involved over time or more closely, they were unlikely to observe how their input was integrated into the effort.	• Survey implementers as to how they incorporated patient feedback (Fehling, webinar, 2016) [52] • Create and use a structured schedule to routinely tell patients how their feedback was incorporated [56, 58] • Explain why alternate decisions were made [56]
8) Engaging patients too late in the implementation effort for their engagement to be useful or realized	Participant Interview 7 described this as trying to “avoid the ‘lasagna problem’… if you have a consultation [with patients] two weeks before a [proposal] is done, the lasagna is already baked, and you can’t take it out of the oven and change it five minutes before.”	• Meet at regular, scheduled intervals (Participant Interview 6) • Engage patients throughout life cycle of project [56] • Use meeting processes to truly make sure partners are equal (Participant Interview 6) • Sustain patient partnerships over time (Participant Interview 6) • Plan special events in advance [59]

*Note.* Recurring challenges 1 and 2 are described with solutions in more depth as examples in the text of the manuscript—abbreviated versions are provided in this table. Each data source is listed after the solution. Some solutions were meant to address more than one challenge—they are not duplicated in Table 3, however

### Recurring challenge 1: lack of processes to communicate equitably among all people

Implementers described a dearth of informal and formal processes to ensure equitable communication between patients and healthcare professionals within existing power differentials. Equitable communication would consist of comfort sharing and receiving feedback bidirectionally, a nonjudgmental atmosphere, and shared opportunities for all to speak and listen. In one webinar, Asch [54] emphasized the difficulty healthcare professionals face in learning “*to speak with [patients] effectively about research*” and implementation. Communication processes were also needed to ensure the ‘right’ people for an issue could share feedback at their comfort level and that others could understand this feedback.

### Promising solutions to a lack of processes to communicate equitably

Solutions for ensuring equitable communication centered on behaviors for patients or healthcare professionals.

#### Behaviors for Patients

Patients might take introductory research workshops or short courses, as they did in one effort, “*so when they were at the table with researchers, they could have conversations*” (Interview participant 6, 2019). Another patient-led solution was to create rules of engagement for meetings, suggesting:

“…*one of those rules is confidentiality in the meeting and a right to pass…not only the right to pass if they don’t feel like mentioning or talking about a topic that would make them uncomfortable or disclosing any personal health information, but it’s also the right to pass somewhat on the question the [implementers] bring to the meetings and bring up other questions that they think might be more valuable*” (LaChapelle, webinar, 2017) [53].

#### Behaviors for healthcare professionals

One implementer recommended asking professionals to make their presentations more patient-friendly, emphasizing the need to “*spell out acronyms and do not use acronyms; also use layperson terms, not medical terms*” (Fehling, webinar, 2016) [52]. Another implementer (who was also a patient) acknowledged that even having patients present changed the dynamic of communication: “*Sometimes our [patient] influence is just our presence – they change the way the information is shared, and the way they operate because a patient is there*” (Interview participant 5, 2019). Another presenter described this shift to consider:

“*What [patient] groups tend to want is a lot more in the direction of practicality. What program or policy does the [healthcare system] need to do? How is this going to affect the program or policy? …You have to know who the individual across the table from you and figure out what it is that they want and they know*.” (Asch, webinar, 2018) [54].

During the meetings, some implementers found it helpful to explicitly state that patients were experts there and their voice was highly valued, thereby empowering patients to educate the rest of the group on topics related to their lived experience (Barger et al.,2019) [47]. Further instructions for the person leading meetings were described:

“*Be versed in group dynamics, allowing suggestions and comments to be made that are not always ‘pro healthcare.’…There would certainly be times not only for general roundtable discussion but also for direct questioning of less-vocal group members to ensure the broadest possible discussion of opinions.*” (Angstman, literature article, 2009) [41].

Angstman et al. [41] also suggested limiting the size of groups to facilitate better communication “*to approximately 15 members. A larger group may be intimidating and may limit discussion*.”

### Recurring challenge 2: recruiting a diversity of patients representative of actual patients served

Across engagement levels, many implementers described difficulties recruiting patients with a demographic makeup consistent with actual patients served. For example, one implementer discussed the challenges of engaging U.S. military veterans who were women or racially minoritized to serve on veteran advisory boards. Many data sources pointed to difficulty engaging people burdened by health problems or societal disadvantages such as poverty. For example, in a literature article concerning implementation of asthma interventions, Tapp [44] reported mostly engaging patients with advanced education or careers in healthcare that were not representative of the broader swath of patients living with asthma [60]. Implementers noted that the patients engaged were more homogenous than populations served in their settings.

### Promising solution to recruiting a diversity of patients

One solution suggested in the data we scanned was to recruit a broader diversity of patients by compensating them for their time (Tapp 2017, literature article) [44], and aligning administrative logistics such that they could be paid financially (Fehling 2016, webinar) [52]. Although, financial compensation did not guarantee engagement, as exemplified in one example in which patients did not respond regularly to electronic messages or paper letters requesting feedback even after being oriented, agreeing to engage, being paid for their time (Participant Interview 3). Other solutions to ensure a diverse representation of patients included providing accommodations for their needs, such as providing childcare during meetings, convenient meeting times (which will vary based on life context), and transportation to meetings (Pérez Jolles, 2017, literature articles) [43].

## Discussion

Through an environmental scan in the U.S., we identified 27 examples of patient engagement in healthcare service delivery implementation activities. Many examples of “how” implementers are doing this work included assembling patients in groups (“councils” or “steering committees”) to advise implementation or incorporating patients into existing implementation teams as participating members. There are certainly other ways to engage patients in design/delivery of implementation activities, including training them to facilitate change in healthcare systems, [61] sampling their viewpoints repeatedly through surveys, interviews, [49] or practice run-throughs as mock patients before, during, and after healthcare professionals make changes in clinical practice. Implementers described eight recurring challenges. The two most frequently identified challenges were: (1) a lack of processes that allowed for equitable communication among all people and (2) difficulty recruiting a diversity of patients representative of those served in the healthcare setting. In addition, we matched promising solutions described by implementers to recurring challenges. Implementers rarely specified if they were engaging a population experiencing healthcare inequities.

We compared examples in this scan to five patient engagement implementation strategies recommended by experts through a consensus process [9]. Most examples from this scan fell under “involve patients and family members,” a strategy rated by experts as very important *and* feasible. Interestingly, none of the examples in this scan were intentional to “increase demand” or “use mass media”— two strategies rated by experts as the *least* feasible [9].

Regarding the intensity of engagement, half the examples in our scan involved medium intensity level of engagement (“coordination”), such that implementers worked with patients to obtain unidirectional feedback on implementation but did not collaborate in ways such as paying patients, empowering them to make decisions, or asking them to assist with or lead tasks. This finding contrasts a systematic review of global examples, most outside the U.S., in which implementers frequently used higher intensity levels of engagement, including collaboration as partners and co-design of healthcare service delivery [22]. This is important because existing literature suggests that different intensities of engagement may yield different outcomes. In one study of patient engagement in primary care redesign across Ireland, researchers found activities similar to or less intensive than coordination (e.g., information events, one-time consultation with healthcare teams) to be more feasible than higher-intensity engagement. Engaging patients with higher intensity (e.g., integrating patients on primary care teams) proved more difficult, and its usefulness was less clear [62]. And yet, in a global systematic review, authors concluded that unidirectional patient engagement (e.g., consultation) usually led to more discrete products, like a toolkit. In contrast, collaborative and co-created patient engagement led to changes in the care process or structural outcomes [22]. Lower-intensity engagement may be more feasible. Yet, such engagement may produce outcomes with lower impact.

One implication from our findings is that the intensity and process of engaging patients fall on a continuum, [39] and there may not be one ideal intensity level for implementation efforts. Only one example in this scan used the most intensive community-based participatory research (CBPR) approach in implementation activities, emphasizing capacity building, equitable distribution of finances, co-ownership, and having patients collect and analyze information. A CBPR approach to implementation requires significantly more time and resources than coordination and will not always be feasible for implementers or patients. Indeed, Ramanadhan and colleagues suggested that implementers and patients evaluate their goals for an implementation activity to select the optimal patient engagement level [6]. It might be ideal to offer choices for how intensely any person wants and can engage.

The challenges we identified to patient engagement in U.S. implementation activities were consistent with and expanded upon barriers in existing literature from Canada, [63] the U.K., [22] and another U.S. institution [64]. This suggests that some challenges encountered across the U.S. are similar to those in other jurisdictions, despite structural differences between the U.S. and other countries in political and economic regulation of healthcare. Because some challenges are similar, implementers in any region can learn from those in other places. Some countries outside the U.S. appear to have long histories of patient engagement in implementation and higher intensity patient engagement [22, 61] and, thus, a wider range of solutions to those challenges than those identified in this scan [22, 65]. For example, these efforts are supported by financial funding in healthcare systems in Ireland, [62] Canada, [20] and the U.K [19]. Yet, even in countries with strong policy and financial support for patient engagement, variations in strategies and terminology and organizational structure and culture can lead to confusion [19, 62, 66]. Researchers found a prior history of engagement with patients promoted more substantial organizational changes—the longer places engaged with the same community groups or patients, the easier it was to build on existing relationships for ongoing change [62]. Situating our results from the U.S. within the global context of what is necessary for successful patient engagement in implementation activities, it appears two “levels” may be essential. The first level is that structural factors support patient engagement in implementation activities, such as organizational funding, personnel training, and policy support, and these are necessary alongside best practices at the interpersonal level between patients and professionals, such as inclusive recruitment strategies, equitable communication practices, and early engagement processes.

One specific challenge we identified was that implementers could not always engage patients burdened by societal barriers and experiences of healthcare disparities, consistent with findings elsewhere [64]. Engaging populations experiencing healthcare disparities means overcoming societal disadvantage and mistrust experienced by these populations and perpetuated by unjust structures in the U.S [64]. Implementers need to be proactive, creative, and intentional about engagement strategies for patients experiencing disparities. Most examples in this scan did not report patients’ demographic characteristics or lived experiences, making it difficult to determine whether they involved patients experiencing disparities. The absence of such critical information was also documented in a systematic review of patient engagement in implementation activities in the U.K [19]. Across jurisdictions, there might be less attention to inequities, power differentials during engagement, and strategies to overcome barriers for those experiencing disparities. Implementers might need to interrogate privileges and biases they bring to engagement encounters with people impacted by health disparities, akin to cultural humility [57]. A future research question is whether a more robust use of patient engagement in implementation activities reduces healthcare disparities.

### Limitations

While we scanned literature and implementers from any U.S. healthcare system, within that, we scanned some data from webinars narrowed to one system. Although this was a thoughtful choice to extend a recent systematic review of global literature, [22] this approach limits the external validity of our findings. Interviewing a convenience sample of implementers also limits our ability to be comprehensive. Finally, we did not identify which implementation strategies were used (e.g., financial incentives, changing physical structure).

## Conclusion

Despite patients being engaged in numerous implementation activities across healthcare settings, there are no clear guidelines on how to facilitate their participation or what level of engagement is recommended in the U.S. Our findings offer implementers ideas for how to engage patients in design/delivery of implementation activities, including considerations for more “light touch” or less intensive engagement depending on patient preference, resources, or prior implementer skills, and also more intensive engagement which might result in meaningful, patient-centered shifts on a larger scale. There may not be one ideal level of intensity, which policies on patient engagement in implementation should consider, although this is a topic for hypothesis testing in future research.

Compared to other countries, like the U.K., with longer histories and more structural support for patient engagement in implementing changes in healthcare service delivery, implementers in the U.S. appear to have similar challenges in recruitment, retention, and meaningful engagement. Thus, one practice implication is that implementers in the U.S. can learn from other countries that appear to have more varied solutions and experiences responding to challenges. However, across jurisdictions, no countries appear to have a robust focus or strong support for patients experiencing disparities to improve health equity.

One funding and policy suggestion is that patient engagement should be considered as another piece of the implementation puzzle in U.S. healthcare, along with implementation strategies targeting clinic staff, leadership, organizational structures, and policies. Patient engagement in implementation activities can potentially reduce inequities through local tailoring at the clinic-level that is more patient-centered and enhanced end user buy-in for interventions [4, 25]. If prior experience with engagement makes it easier to engage patients in the future, [62] leading to change, then the history of health inequality in the U.S [56, 58, 59] means we must double-down on efforts to engage patients experiencing disparities to move toward health equity.

### Supplementary Information

**Additional file 1. **Implementer Survey and Semi-structured Interview Questions. This is a document showcasing the survey questions we used in interviewing implementers for the scan

**Additional file 2. **Coding Template of Patient Engagement Activities in Implementation, Systems Redesign, or Quality Improvement. This is a document of a blank coding template used to extract data from each case we identified in the environmental scan

## Data Availability

The datasets used and/or analysed during the current study available from the corresponding author on reasonable request. Please contact the first, second, or last author.

## Abbreviations

CINAHL: Cumulative Index of Nursing and Allied Health Literature
CBPR: Community-based participatory research
PCORI: Patient-Centered Outcomes Research Institute
U.K.: United Kingdom
U.S.: United States
VHA: Veterans Health Administration
