# Supplementary material for: Challenges and promising solutions to engaging patients in healthcare implementation in the United States: an environmental scan
Source: BMC Health Serv Res. 2024 Jan 4;24:29. doi: 10.1186/s12913-023-10315-y (PMC10768202; doi:10.1186/s12913-023-10315-y)
Supplement: Supplementary file 1 — Additional file 1. Implementer Survey and Semi-structured Interview Questions. This is a document showcasing the survey questions we used in interviewing implementers for the scan [file 12913_2023_10315_MOESM1_ESM.docx]

Additional File 1. Implementer Survey and Semi-structured Interview Questions. This is a document showcasing the survey questions we used in interviewing implementers for the scan.

*Thank you for filling out this document! Write as little or much as you like. If we have questions, we will follow up with another e-mail or phone call. We will compile your submission with others and de-identify any specifics.*

1. How do you engage consumers in implementation? Consumers are patients or end users of an intervention, not other stakeholders, such as hospital leaders, providers, or staff.
2. Have you engaged patients/consumers in implementation, systems redesign or quality improvement?
   1. What structures or tools or practices have you used to do this work? Please cite or send any references.
   2. Were any of the consumers you engaged people who suffer health disparities?

Health disparities are significant differences in access to, quality or, or outcomes of healthcare, due to societal disadvantage. E.g, you might implement Hepatitis C Virus medicine among Black and African American people who are more likely to have Hepatitis C Virus in the United States.

If yes; what did you need to do to promote meaningful consumer engagement among these consumers?

1. Think of one or two examples of engaging consumers in implementation. Below are fill-in-the-box questions to assess some of the details about the change in clinical care you were trying to influence. Use bullets or numbering if you list two examples.

| **Detail** | **Example** | **Fill-in-the-box** |
| --- | --- | --- |
| What were you trying to implement or improve? | Evidence based psychotherapy |  |
| Who needed to change their behavior to implement something (e.g., providers)? | Mental health providers (e.g., psychologists, social workers) |  |
| Who were the consumers? | Two patient advisors who had received evidence-based psychotherapy |  |
| What were the roles of patients or other consumers in implementation? | Patient advisors served as co-investigators and had decision making capabilities regarding key aspects of the implementation research |  |
| At what kind of sites and how many did this occur (e.g., 1 clinic; 3 hospitals)? | 4 mental health clinics in 1 state – 2 were private practice, 2 were hospital-based |  |
| Did these sites belong to a larger system? | 2 belonged to a state healthcare system |  |

1. What are some problems you encountered in engaging consumers in implementation? Describe the top 3 issues below.

| **Problem** | **Was it able to be solved?** | **How did you solve it?** |
| --- | --- | --- |
|  |  |  |
|  |  |  |
|  |  |  |
