# Supplementary material for: Challenges and promising solutions to engaging patients in healthcare implementation in the United States: an environmental scan
Source: BMC Health Serv Res. 2024 Jan 4;24:29. doi: 10.1186/s12913-023-10315-y (PMC10768202; doi:10.1186/s12913-023-10315-y)
Supplement: Supplementary file 2 — Additional file 2. Coding Template of Patient Engagement Activities in Implementation, Systems Redesign, or Quality Improvement. This is a document of a blank coding template used to extract data from each case we identified in the environmental scan [file 12913_2023_10315_MOESM2_ESM.docx]

Additional File 2. Coding Template of Patient Engagement Activities in Implementation, Systems Redesign, or Quality Improvement. This is a document of a blank coding template used to extract data from each case we identified in the environmental scan.

| Patient Engagement Level | Patient engagement example (what they do)  What structures or tools are used and for what purpose (e.g., groups, listserv?) | Problems?  Solutions or resources? | Innovation; recipients; inner contexts; outer context; societal context | Are they engaging populations who suffer health disparities? |
| --- | --- | --- | --- | --- |
| Outreach: Implementers try to reach patients |  |  |  |  |
| Education (Implementers educate patients) |  |  |  |  |
| Coordination: Implementers gather patients to give consultation but with no decision-making power. Patients do not give help. |  |  |  |  |
| Cooperation: Patients help implementers carry out some task, instead of just giving advice, some decision-making power. Patients gain insight into research. |  |  |  |  |
| Collaboration: Implementers and patients design, implement, and interpret findings. All benefit. Patients have equitable power. |  |  |  |  |
| Patient-Centered: Patients dictate decisions and implement things and interpret findings. Implementers contribute expertise, but patients make all decisions about approaches. Patients can engage in partnerships with equitable power for governance. |  |  |  |  |
| Community-Based Participatory Research: Population health approach to the patient-centered engagement model. Trust among partners, respect for each partner’s expertise and contributions, mutual benefit among partners, and a community-driven partnership with equitable and shared decision-making. |  |  |  |  |
